# Study on the Seismic Performance of Steel Tube-Reinforced Concrete Columns After Fire on One Side

**DOI:** 10.3390/ma18091975

**Published:** 2025-04-26

**Authors:** Chong Tang, Yanhong Bao, Yang Yu

**Affiliations:** 1 Department of Civil Engineering and Water Resources, Qinghai University, Xining 810016, China; cetangc@163.com; 2 Centre for Infrastructure Engineering, Western Sydney University, Sydney 2000, Australia

**Keywords:** steel tube-reinforced concrete columns, seismic performance, post-fire behavior, single-side fire exposure, parameter analysis, numerical simulation

## Abstract

To investigate the seismic performance of steel tube-reinforced concrete (ST-RC) columns after fire on one side, this study employs numerical simulation and theoretical analysis methods. A numerical analysis model of ST-RC columns post-fire is established using ABAQUS to simulate and analyze their seismic performance under cyclic loading. The characteristics of the hysteresis curves of ST-RC columns after fire on one side under cyclic loading are described in detail. Comparisons are made between the skeleton curves, ductility, stiffness degradation, and energy dissipation capacity of ST-RC columns under three conditions: unexposed to fire, exposed to fire on all sides, and exposed to fire on one side. Finally, multiple influencing factors, including heating time, slenderness ratio, section size, core area ratio, external concrete strength, reinforcement ratio, and load ratio, are selected for parametric analysis of the ductility coefficient, stiffness, and viscous damping coefficient. Mathematical formulas for the ductility coefficient, stiffness, and viscous damping coefficient of ST-RC columns after fire on one side under cyclic loading are derived through regression analysis. The results show that the seismic performance of ST-RC columns is attenuated after fire on one side, and the ductility and initial stiffness of ST-RC columns decreases by 5.62% and 24.69%, respectively, compared with those without fire. The energy dissipation capacity of the ST-RC column increases significantly when it enters the plastic deformation stage under the action of reciprocating load.

## 1. Introduction

**Figure 1:**
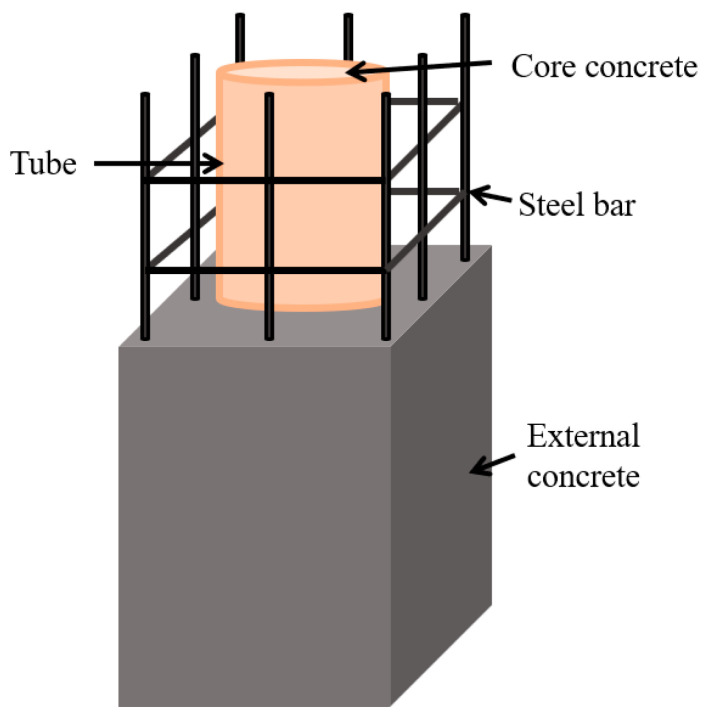
Specific composition of the ST-RC column.

Steel tube-reinforced concrete (ST-RC) columns represent a novel type of composite structural member, renowned for their exceptional fire resistance [1,2,3,4] and seismic performance [5,6]. They are widely utilized in high-rise buildings [7,8], such as the Postal Hub Building in Liaoning, China, and the Nordic Financial Centre in Shenzhen, China. These columns are constructed by binding reinforcement around a concrete-filled steel tubular (CFST) column and subsequently casting additional concrete, as illustrated in Figure 1. To date, numerous scholars have conducted extensive research on the fire resistance and mechanical properties of ST-RC columns under fire conditions [9,10,11], establishing a comprehensive theoretical foundation.

In real-life scenarios, fire sources are typically extinguished promptly. However, the performance of structural members may deteriorate after fire exposure, necessitating an analysis of their post-fire cooling behavior if they are to be reused. To address this, Xiang [12] investigated the residual bearing capacity of ST-RC columns after fire exposure. He concluded that repair and strengthening measures are essential for reusing fire-damaged members and proposed formulas for calculating the residual bearing capacity and axial stiffness of ST-RC columns after cooling. In regions with high seismic fortification intensity, the assessment of fire-damaged building structures requires not only an evaluation of residual bearing capacity but also a thorough analysis of seismic performance. The slenderness ratio has been shown to significantly influence both the fire resistance and seismic performance of structural members, as demonstrated by Wu [13] and Yao [14]. Building on these findings, Wang [15] selected an appropriate slenderness ratio to conduct a detailed study on the seismic performance of ST-RC columns after exposure to fire on all sides. The study revealed that ST-RC columns maintain excellent seismic performance under cyclic loading, even after fire exposure.

**Figure 2:**
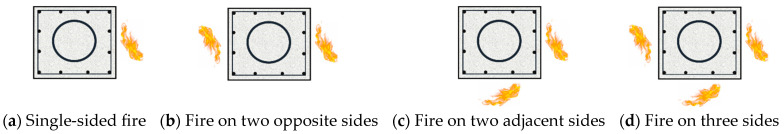
Specific case of non-uniform fire.

Although the aforementioned scholars have conducted extensive research on various properties of ST-RC columns, they have not addressed the scenario of non-uniform fire exposure. During a fire, columns are not always subjected to fire on all four sides; instead, non-uniform fire exposure is more common, as illustrated in Figure 2. Many researchers have already recognized this issue. Lama [16] performed nonlinear analysis and design of CFST columns under non-uniform fire exposure, revealing that for certain slender columns with a load ratio below 0.47, low fire resistance ratings fail to meet design requirements. This underscores the importance of selecting an appropriate load ratio. Meng [17] investigated the fire resistance of steel tube-filled reinforced concrete columns but did not develop a method to predict their fire resistance under non-uniform fire conditions. Mao [18], after selecting an appropriate load ratio, studied the fire resistance of such columns and proposed an improved calculation method to predict their fire resistance. Guo [19] also developed a 3D finite element model to predict the ultimate bearing capacity of CFST columns under non-uniform fire exposure. While these studies focused on non-uniform fire exposure, they did not examine the post-fire performance of structural members. Lyu [20] and Xu [21] investigated the seismic performance of composite members after non-uniform fire exposure. However, research on the seismic performance of ST-RC columns following non-uniform fire exposure has yet to make significant progress.

Among all types of fire exposure, it is generally believed that single-side fire exposure has the least impact on the structure. The materials piled up on one side of the column caught fire and were not extinguished in time. At this point, only one side of the column was exposed to fire. The structure, after being exposed to fire, needs to undergo rigorous performance analysis and cannot be judged merely by imagination. Building on the aforementioned context, this study investigates the seismic performance of ST-RC columns after fire exposure on one side. The primary focus is on analyzing the hysteresis curves, skeleton curves, ductility, stiffness, and energy dissipation capacity of ST-RC columns after fire exposure on one side. These characteristics are systematically compared with those of columns that were unexposed to fire and those exposed to fire on all sides. Finally, key parameters are selected for parametric analysis, and mathematical formulas for the ductility coefficient and viscous damping coefficient of ST-RC columns after fire exposure on one side are derived through regression analysis.

## 2. Establishment of Finite Element Model

### 2.1. Stress Analysis Model

The thermal models for steel and concrete are adopted from Lie [22], which have demonstrated excellent accuracy in temperature field calculations. The steel material model utilizes the kinematic hardening model proposed by Han [23], effectively mitigating the Bauschinger effect. For the compressive behavior of core and external concrete, the models proposed by Lin [24] and Tan [25] are employed, respectively. These models have proven effective in post-fire repair and strengthening applications. The tensile model is based on the formulation proposed by Hu [26], which accounts for the natural cooling process and the maximum fire temperature. This model comprehensively considers the loss of moisture in concrete after high-temperature exposure and the increase in internal porosity, resulting in reduced post-fire strength. The aforementioned material constitutive models incorporate material damage by considering the maximum fire temperature in seismic damage modeling. For simulations under normal temperature conditions, the constitutive models are derived from the corresponding material models at 20 °C.

In this study, the concrete plastic damage model is employed to simulate the stiffness and strength degradation of ST-RC columns under seismic conditions following fire exposure. This model effectively captures the mechanical behavior of concrete under cyclic loading. The damage coefficients proposed by Zeng [27] and Li [28] are applied to the external and core concrete, respectively, to accurately represent the behavior of concrete both before and after crack closure.

### 2.2. Modeling Method

When simulating fire effects, the component model adheres to the ISO-834 [29] standard for temperature rise and fall. Heat is transferred to the component’s surface through thermal convection and radiation. The thermal radiation and convection coefficients are adopted from ECCS [30]. The comprehensive radiation coefficient (*ε*) for both the fire-exposed side and the back side is set to 0.5, while the thermal convection coefficients (*α*) are set to 25 W/(m^2^·°C) and 9 W/(m^2^·°C), respectively. The contact between different parts of the component is constrained using “Tie” constraints to ensure efficient heat transfer. Concrete is modeled using DC3D8 elements, the steel tube using DS4 elements, and the reinforcement using DC1D2 elements.

**Figure 3:**
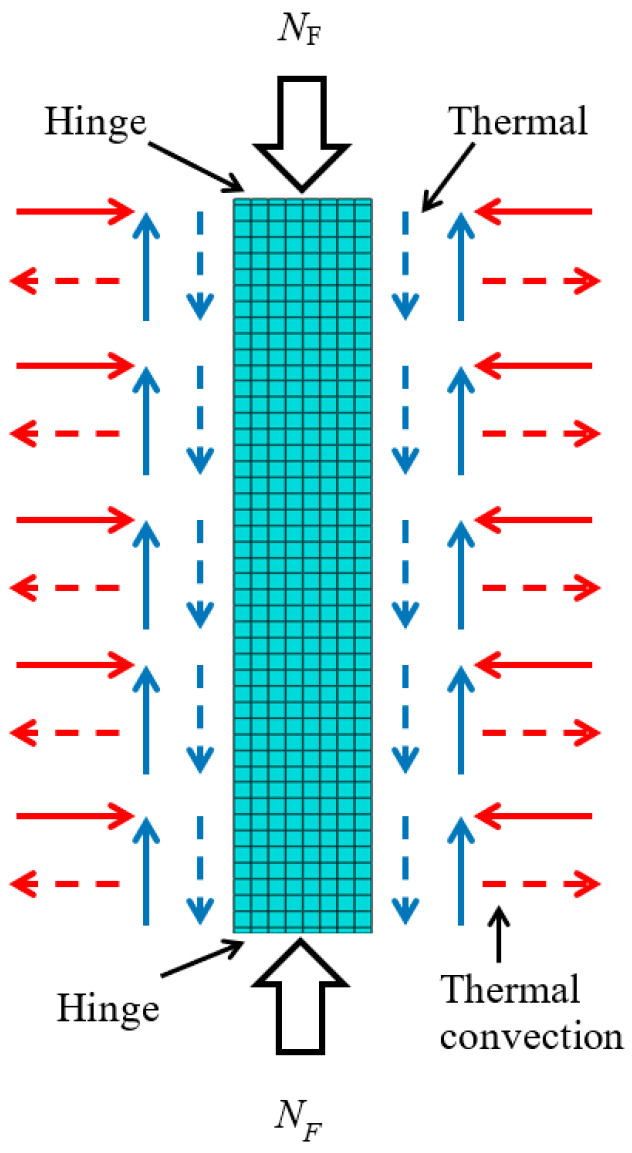
Finite element model of ST-RC columns.

After fire exposure, the building structure is subjected to low-cycle horizontal loading simulations. It is assumed that no slippage occurs between the reinforcement and concrete. There is surface contact between the external concrete and the steel, as well as between the steel and the core concrete. The interaction between the tube and concrete is modeled as hard contact in the normal direction and frictional contact in the tangential direction, with a friction coefficient of 0.6 [30]. The reinforcement is embedded in the external concrete using the “Embedded” constraint method. The axial load at the top of the column is applied as a concentrated force, calculated as the residual bearing capacity of the component after fire exposure multiplied by the axial compression ratio. The top of the column is constrained to allow only axial movement, while the mid-span is restricted to allow only lateral movement. Concrete and the steel tube are modeled using C3D8 elements, and the reinforcement is modeled using T3D2 elements. A mesh sensitivity analysis is conducted to determine the optimal meshing strategy, with the final mesh size set to 10–20 mm in the length and width directions and 1–2 times that size in the height direction. The finite element model is shown in Figure 3.

### 2.3. Verification of Finite Element Model

**Figure 4:**
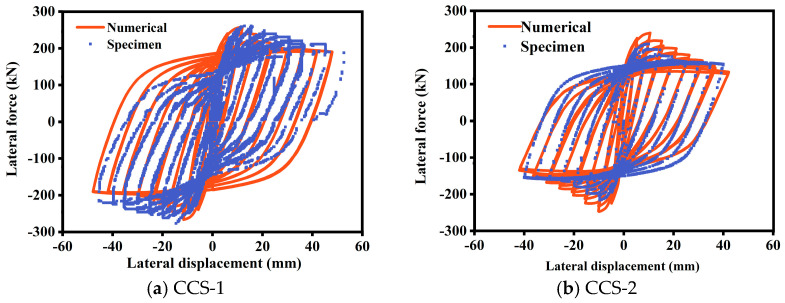
Seismic comparison of calculated and tested results of specimens.

**Figure 5:**
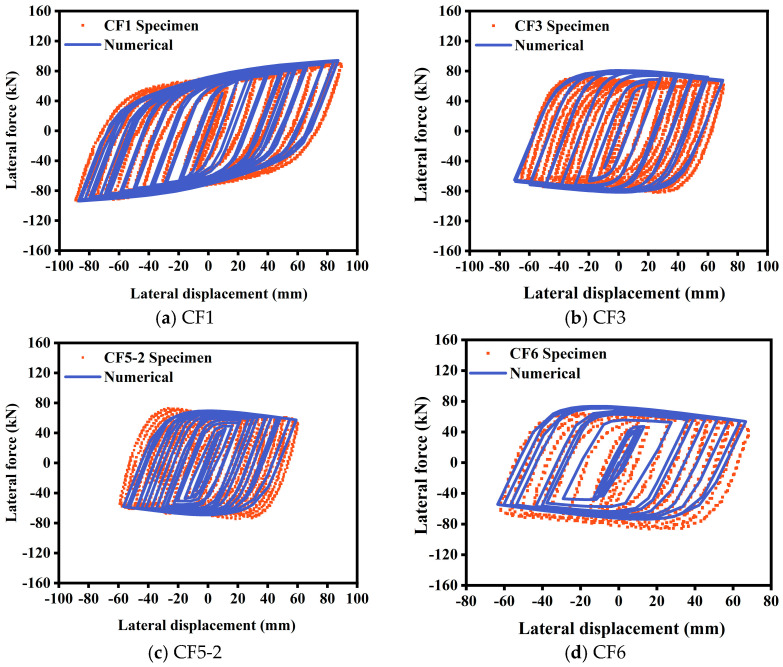
Comparison of calculated and tested hysteresis curves of specimens after fire.

Due to the lack of experimental data on low-cycle reciprocating tests of ST-RC columns after fire exposure, this paper indirectly validates the results by simulating seismic damage tests of ST-RC columns and post-fire seismic damage tests of CFST columns in Abaqus (6.12, Dassault Systèmes, PAR, FR). The test parameters are listed in Table 1, and the comparison between the calculated results and the experimental results is shown in Figure 4 and Figure 5.

Figure 4 presents a comparison between the experimental and calculated hysteresis curves from seismic tests on ST-RC columns under normal temperature conditions. The curves exhibit a high degree of agreement, with excellent correspondence in peak values for each loading cycle and similar horizontal bearing capacities. This indicates that the simulation results are accurate and reliable.

Figure 5 presents a comparison between the experimental and calculated hysteresis curves from seismic tests on CFST columns after fire exposure. The calculated results show excellent agreement with the experimental results, with accurate correspondence in peak values for each loading cycle. The pinching effect, typically observed in bow-shaped hysteresis curves, is less pronounced in the calculated spindle-shaped hysteresis curves, resulting in a negligible impact.

**Figure 6:**
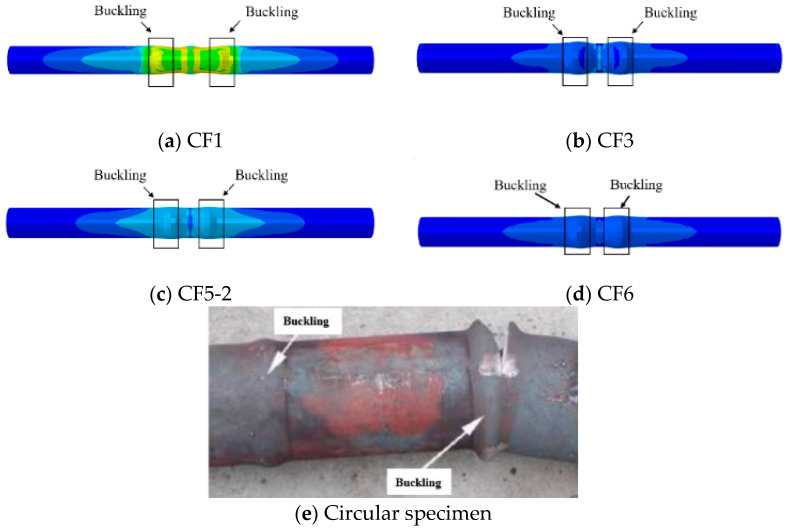
Comparison of damage patterns of specimens.

Figure 6 compares the failure modes of CFST columns obtained from experiments and numerical calculations. It is evident that both the experimental and calculated components exhibit bulging, thereby validating the accuracy of the modeling approach. This modeling method can be effectively applied to analyze the seismic performance of ST-RC columns following fire exposure.

## 3. Seismic Mechanism Analysis After Fire on Single Side

**Figure 7:**
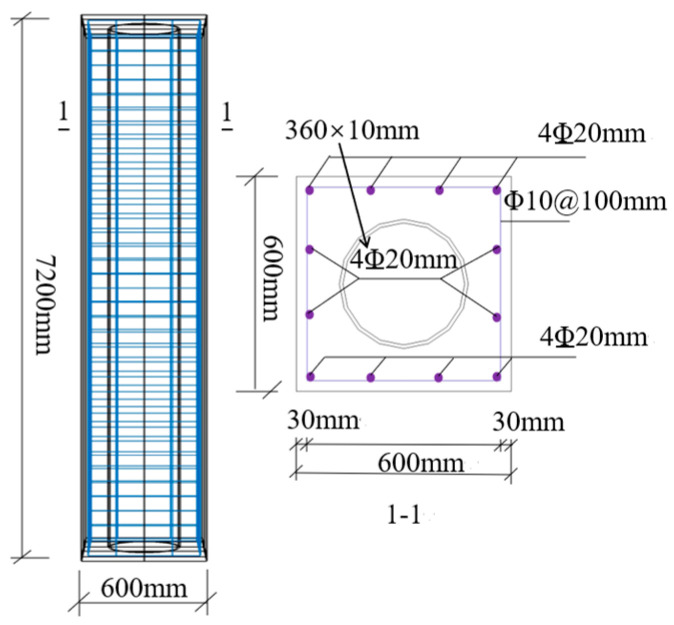
Calculation model of ST-RC columns.

**Figure 8:**
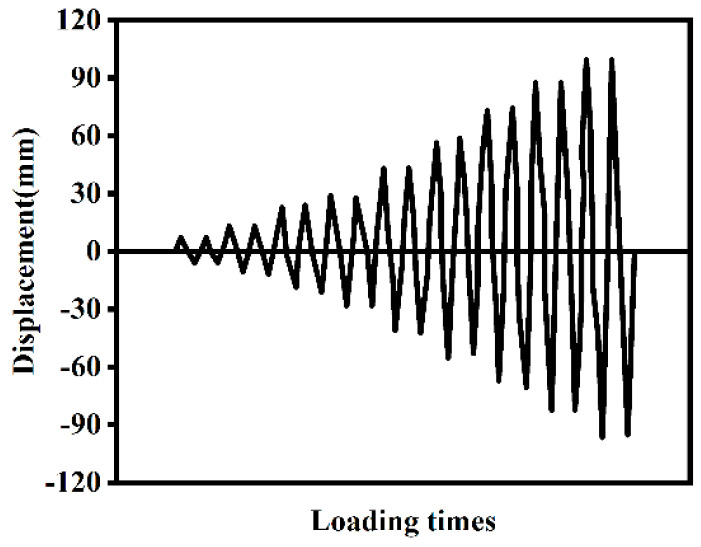
Cyclic load loading diagram.

The numerical analysis model of the ST-RC column is established in accordance with the standards GB 50936 [31] and T/CECS [32] to enable a more precise analysis of the seismic performance of ST-RC columns after fire on one side. The specific parameters are detailed in Table 2, and the constructed model is illustrated in Figure 7. The heating time, based on Tang [33], is set to 120 min. The loading protocol, as depicted in Figure 8, adheres to the guidelines of JGJ/T 101-2015 [34]. For displacements less than 20 mm, each loading increment is 5 mm with two cycles; for displacements exceeding 20 mm, each loading increment is 10 mm with two cycles, continuing until failure.

*B* is the section side length, *D* is the inner diameter of the steel pipe, *t* is the thickness of the tube, and *L* is the column length. The thickness of the protective layer is 30 mm. *e*′ = e/*r*_0_, where *r*_0_ = *B*/2. *ρ*_b_ is the reinforcement ratio. *n* = *N*_0_/*N*_u_(t), where *N*_0_ is the axial force applied, and *N*_u_(t) is the ultimate bearing capacity of the ST-RC column after fire with a heating time of t minutes. *α*_sc_ is the core area ratio, and the value is the core concrete section area divided by the total section area.

### 3.1. Hysteresis Curve

**Figure 9:**
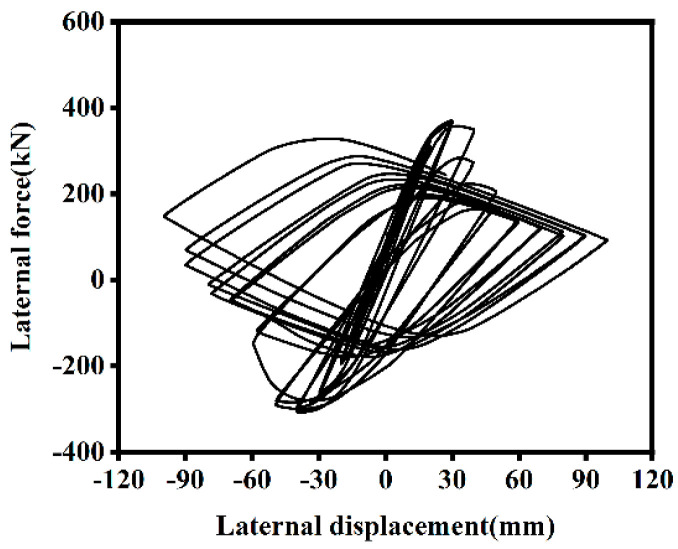
Hysteresis curve of ST-RC columns.

Hysteresis curves provide valuable insights into the strength, stiffness, ductility, and energy dissipation capacity of a structure. Figure 9 illustrates the relationship between horizontal force and loading displacement for ST-RC columns under cyclic loading after fire on one side. The hysteresis curves of the components after fire on one side are spindle-shaped and relatively full, indicating that the components primarily undergo bending deformation. The large enclosed area of the curves reflects the strong plastic deformation capacity and excellent seismic performance of the components, both at normal temperature and after fire on one side.

Before yielding, the hysteresis loops are small and spindle-shaped, indicating that the specimens are in the elastic stage. The slope of the loading segment remains consistent, and the residual displacement during unloading is minimal, with no significant degradation in ductility, strength, or stiffness. As loading continues, the enclosed area of the hysteresis loops increases, and the energy dissipation capacity of the components improves, marking the transition into the elastoplastic stage. When the bearing capacity reaches the peak load of 365.57 kN, the corresponding peak displacement is 29.83 mm. After the specimen reaches the ultimate load, the bond performance between the reinforcement and concrete further deteriorates, leading to a noticeable decline in strength. The components then enter the plastic stage, undergoing plastic deformation, and the enclosed area of the hysteresis loops significantly increases, further enhancing the energy dissipation capacity.

During unloading, the unloading stiffness remains similar to the stiffness in the elastic stage. Part of the cross-section transitions from tension to compression, with the tension zone gradually decreasing and the compression zone expanding. When the external load is reduced to zero, residual strain occurs due to stiffness degradation and residual compressive stress within the component. During reverse loading, the stiffness of the component remains unchanged, but the compression zone of the cross-section continues to expand. As reverse loading enters the elastoplastic stage, the compression zone area of the composite column increases with horizontal displacement, and the outer reinforced concrete experiences plastic damage, leading to stiffness degradation. With repeated unloading and loading cycles, damage accumulates in the outer reinforced concrete, resulting in continuous reductions in strength and stiffness.

**Figure 10:**
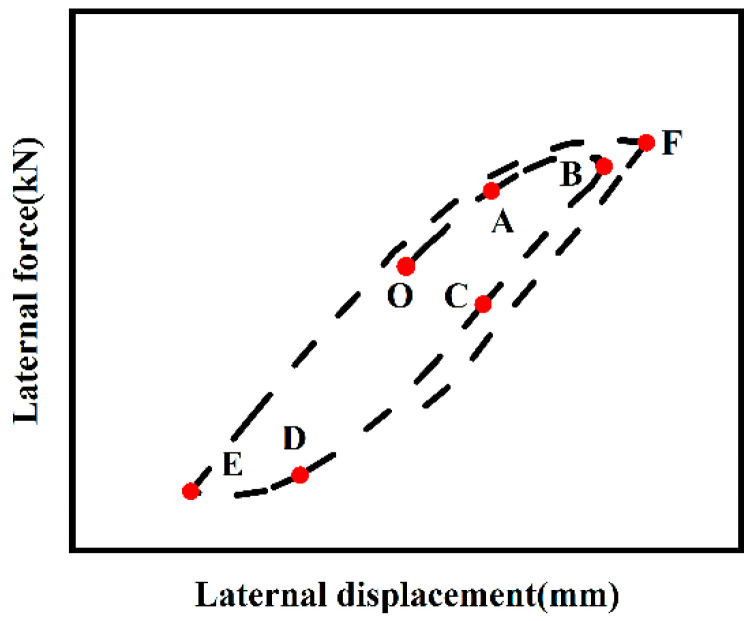
Typical hysteresis curve of ST-RC columns.

Figure 10 illustrates the typical hysteresis curve of an ST-RC column under cyclic loading after fire on one side. Based on reference [15], the curve can be divided into the following six stages:

(1) O-A Stage: The load-displacement curve exhibits a linear relationship, indicating that the component is in the elastic stage.
(2) A-B Stage: After reaching point A, the component begins to enter the elastoplastic stage as the horizontal displacement increases. The stiffness of the composite column also starts to decline.
(3) B-C Stage: Unloading begins at point B. During this process, the unloading stiffness remains similar to the stiffness in the elastic stage. Part of the cross-section transitions from tension to compression, with the tension zone gradually decreasing and the compression zone expanding. When the external load is reduced to zero, residual strain occurs due to stiffness degradation and residual compressive stress within the composite column.
(4) C-D Stage: After point C, reverse loading begins. The stiffness remains unchanged, but the compression zone of the cross-section continues to expand.
(5) D-E Stage: The composite column enters the elastoplastic stage during reverse loading. As the horizontal displacement increases, the area of the compression zone in the cross-section expands, and the outer reinforced concrete experiences plastic damage, leading to stiffness degradation.
(6) E-F Stage: With repeated unloading and loading cycles, damage accumulates in the outer reinforced concrete, resulting in continuous reductions in strength and stiffness.

### 3.2. Skeleton Curve

**Figure 11:**
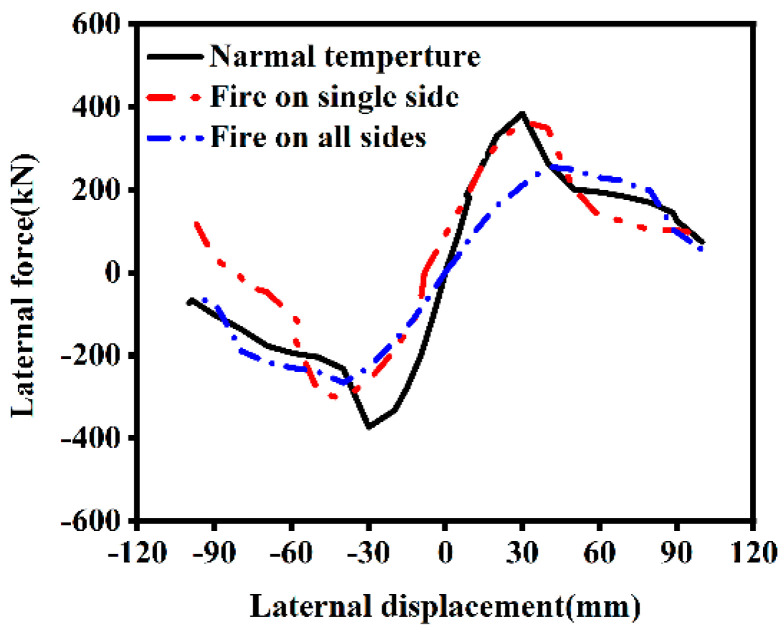
Skeleton curve of ST-RC columns under different fire conditions.

The skeleton curve is derived by connecting the peak points of each hysteresis loop in the hysteresis curve. Figure 11 compares the skeleton curves of ST-RC columns under normal temperature conditions, after fire on one side, and after fire on all sides. From the figure, it is evident that the ultimate displacement of the component after fire on one side is larger, indicating superior ductility.

During the initial loading stage, the peak load and displacement exhibit a linear relationship, and the stiffness shows no significant degradation, indicating that the component is in the elastic stage. As loading progresses, the slope of the curve gradually decreases, and the stiffness of the component declines. When the peak load is reached, the component enters the elastoplastic stage. After yielding, the stiffness of the component significantly decreases due to the influence of residual deformation, ultimately leading to failure.

The ultimate bearing capacity of the ST-RC column decreased by only 4.91% after fire exposure on one side, compared to a 33.36% reduction after fire exposure on all sides. The increase in the number of fire-exposed surfaces weakens the material properties of the component. Single-side fire exposure affects only the material on one side of the component, leaving the overall structural integrity largely intact. As a result, single-side fire exposure has a relatively minor impact on the horizontal ultimate bearing capacity of the component. However, the material properties of steel do not fully recover after cooling from high temperatures, leading to more severe damage on the fire-exposed side. This results in greater overall structural degradation and, consequently, a significant reduction in the ultimate bearing capacity.

### 3.3. Ductility

**Figure 12:**
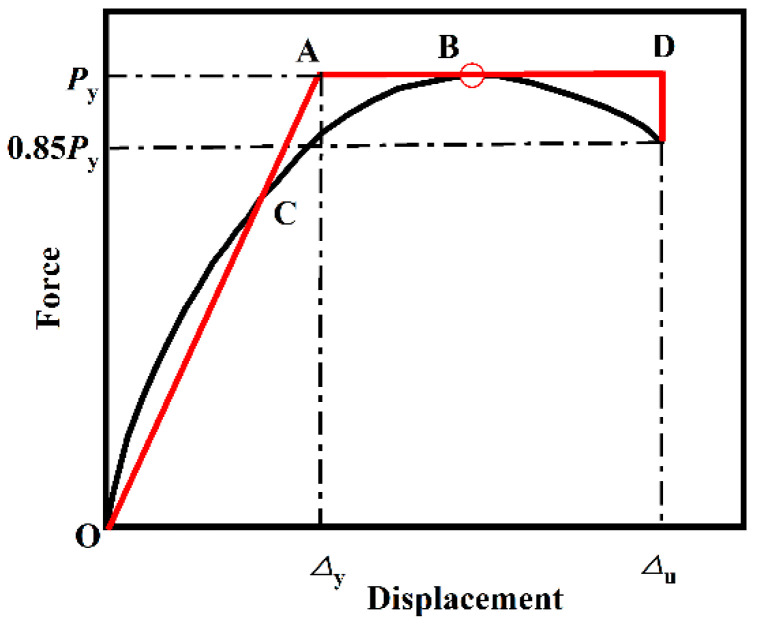
Equal energy method calculation chart.

The ductility of a component reflects its ability to deform under load and can be quantified using the ductility coefficient and the ultimate displacement. When the yield displacement of a component is large, even if the ultimate displacement is also substantial, the ductility coefficient may remain small. Consequently, the ductility coefficient is a reliable metric for comparing the ductility of components only when their yield displacements are similar. In cases where yield displacements differ significantly, the ultimate displacement should be used as the basis for comparison. The ductility coefficient is defined as the ratio of the ultimate displacement to the yield displacement. The yield displacement is determined using the equal energy method, as illustrated in Figure 12, while the ultimate displacement corresponds to the displacement at 85% of the peak load [15].

**Figure 13:**
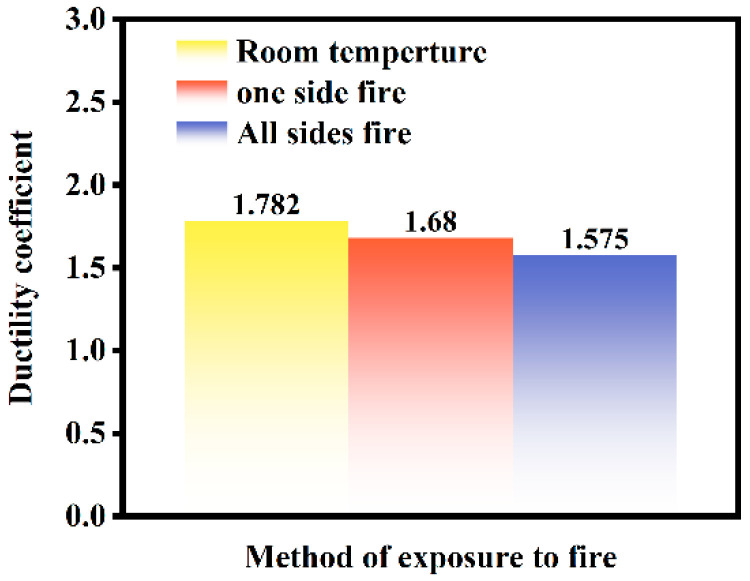
Ductility coefficient of ST-RC columns under different fire conditions.

Figure 13 presents the ductility coefficients of ST-RC columns under cyclic loading under normal temperature conditions, after fire exposure on one side, and after fire exposure on all sides. The ductility coefficients are 1.78, 1.68, and 1.58, respectively. Compared to the ductility coefficient of the component under normal temperature conditions, the ductility coefficients of the components after fire exposure on one side and all sides decreased by 5.62% and 11.24%, respectively. Exposure to fire on one side causes a shift in the geometric centroid or material strength distribution of the member’s cross-section. However, an increase in the fire-exposed surface area leads to a weakening of the material properties, thereby reducing the deformation capacity of the member. This results in a decrease in the ductility coefficient as the fire-exposed surface area increases.

### 3.4. Stiffness Degradation

Stiffness refers to the ability of a structural component to resist deformation under load. It is generally divided into tangent stiffness and secant stiffness. In this paper, secant stiffness is used to represent the stiffness of the composite column under horizontal cyclic loading. The expression is as follows:

(1) $$Ki=\sum_{i=1}^{n}Pij/\sum_{i=1}^{n}\Delta ij$$

In the formula above, *P_ij_* is the horizontal load corresponding to the peak point of the *i* cycle under the J-level loading displacement; Δ*_ij_* is the horizontal displacement corresponding to the peak point of the *i* cycle under the J-level loading displacement; *K_i_* is the secant stiffness during the *i* cycle loading*;* and *n* is the number of cycles.

**Figure 14:**
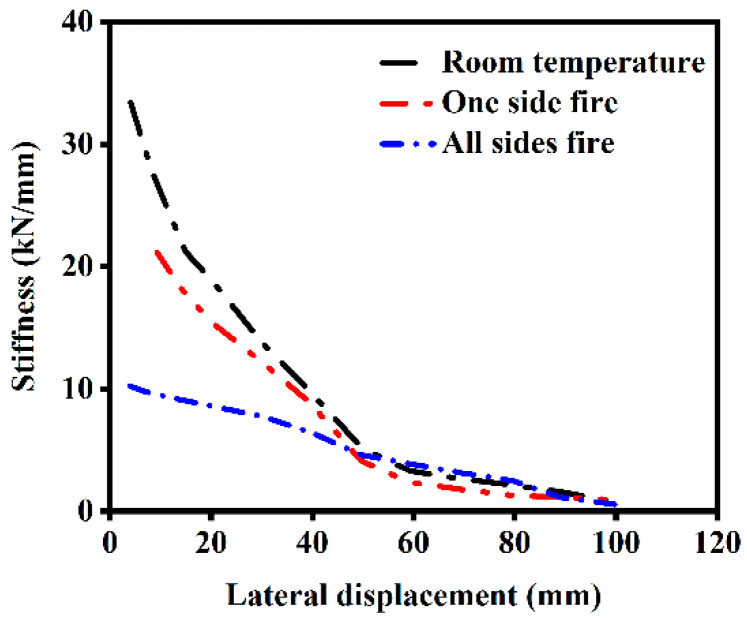
Stiffness of ST-RC columns under different fire conditions.

Figure 14 illustrates the degradation relationship between the secant stiffness of ST-RC columns under cyclic loading and lateral deformation. The following findings can be seen in the figure:

(1) The secant stiffness of components under different fire exposure conditions, including unexposed components, decreases as lateral displacement increases. This is primarily due to concrete cracking and the transition into the elastoplastic deformation stage at larger lateral displacements, which reduces the component’s ability to resist deformation.
(2) As the number of fire-exposed surfaces increases, both the initial stiffness and the secant stiffness at the same lateral displacement decrease. Compared to unexposed components, the initial stiffness of components after single-side fire exposure decreases by 24.69%, while that of components exposed to fire on all sides decreases by 69.37%. This reduction is attributed to the degradation of material properties in both steel and concrete after a fire. Specifically, the elastic modulus of steel cannot fully recover to its pre-fire state after cooling, leading to a reduction in the initial stiffness of the components.

### 3.5. Energy Dissipation Capacity

**Figure 15:**
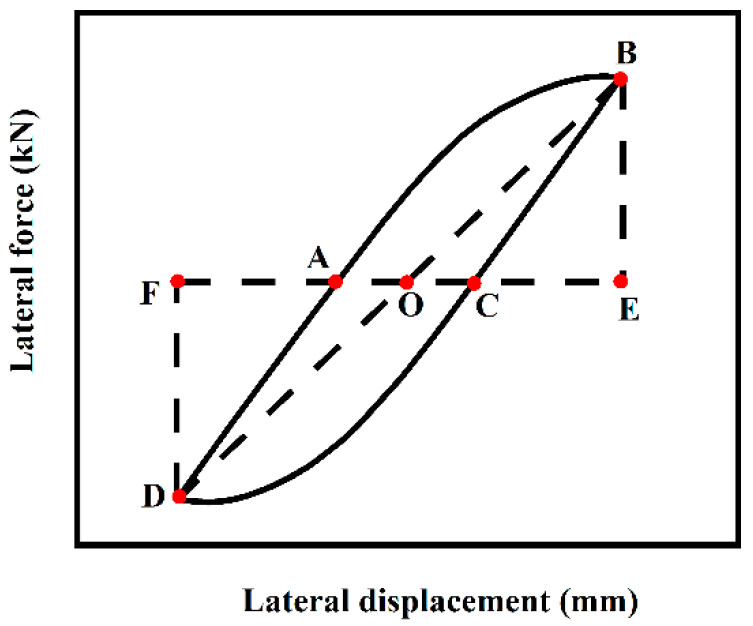
Calculation of energy consumption capacity.

In this paper, the equivalent viscous damping coefficient is used to reflect the energy dissipation capacity of structural members [21]. Its expression is shown below, and the specific area is calculated in Figure 15.

(2) $$h_{e}=\frac{1}{2\pi }\frac{Area(ABC+CDA)}{Area(BOE+ODF)}$$

**Figure 16:**
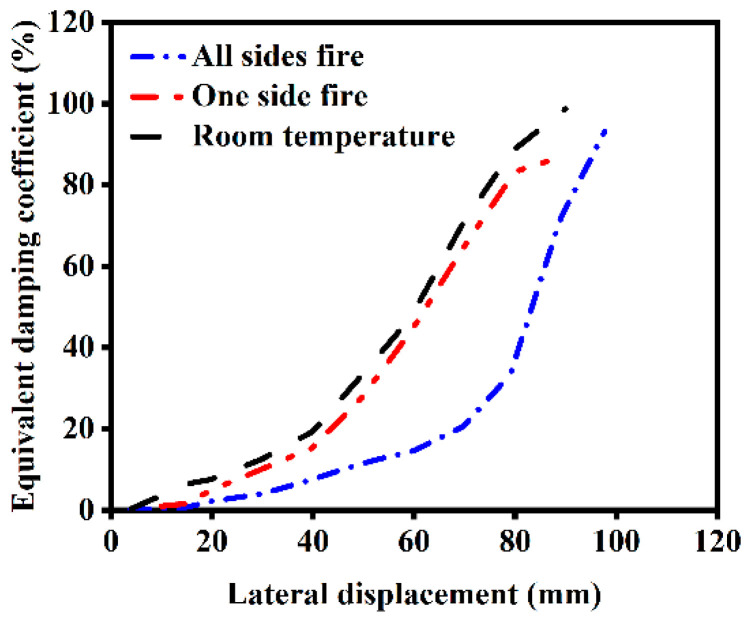
Energy consumption capacity of ST-RC columns under different fire conditions.

Figure 16 compares the viscous damping coefficients of ST-RC columns under normal temperature conditions, after fire on one side, and after fire on all sides. The following findings can be seen in the figure:

(1) In the initial stage of cyclic loading, the structures are primarily in the elastic stage, with small hysteresis loop areas and minimal energy dissipation.
(2) Unexposed components exhibit a higher bearing capacity and relatively fuller hysteresis loops, indicating a superior energy dissipation capacity.
(3) As the number of fire-exposed surfaces increases, the energy dissipation capacity of the components decreases. The energy dissipation capacity of components exposed to fire on one side is slightly reduced compared to unexposed components, while that of components exposed to fire on all sides is significantly reduced. However, when the loading displacement exceeds 80 mm, components exposed to fire on all sides enter the plastic deformation stage under cyclic loading, leading to a notable enhancement in energy dissipation capacity.

## 4. Parameter Analysis of Seismic Performance

In order to analyze the seismic performance of ST-RC columns exposed to fire on one side, a parameter analysis is carried out on different factors related to T/CECS [32], such as heating time (*T*), slenderness ratio (*λ*), section size (*B*), core area ratio (*α*_sc_), external concrete strength (*f*_cu,out_), reinforcement ratio (*ρ*_b_), and load ratio (*n*) within the actual engineering range.

### 4.1. Ductility

Table 3 presents the ductility coefficients of each variable.

#### 4.1.1. Influence of Heating Time on Ductility

When the heating times are 0 min, 120 min, and 180 min, the ductility coefficients of the components are 1.78, 1.68, and 1.58, respectively. As the heating time increases, the ductility coefficient of the ST-RC columns exhibits a decreasing trend, with a 5.72% reduction observed after 120 min of heating. This decline is attributed to the deterioration of steel and concrete materials following fire exposure, which leads to a reduction in the ductility of the components.

#### 4.1.2. Influence of Slenderness Ratio on Ductility

When the slenderness ratios are 8, 12, and 14, the ductility coefficients of the members are 2.0, 1.68, and 1.45, respectively. As the slenderness ratio rises, the ductility coefficient of the ST-RC columns exhibits a decreasing trend. This occurs because, for a constant cross-sectional size, a higher slenderness ratio corresponds to a longer column length and reduced stiffness, leading to a decline in the ductility of the member.

#### 4.1.3. Influence of Section Size on Ductility

When the section sizes are 400 mm, 600 mm, and 800 mm, the ductility coefficients of the components are 1.48, 1.68, and 1.7, respectively. As the section size rises, the ductility coefficient of the ST-RC columns exhibits a decreasing trend. This occurs because with larger section sizes, the reinforcement area increases, leading to a reduced deformation capacity of the member at yield. Consequently, the ductility of the member decreases.

#### 4.1.4. Influence of Core Area Ratio on Ductility

When the core area ratios are 0.166, 0.252, and 0.354, the ductility coefficients of the components are 1.64, 1.68, and 1.81, respectively. As the core area ratio rises, the ductility coefficient of the ST-RC columns exhibits a rising trend. This is because the rise in the area of the inner steel tube and core concrete enhances the ductility of the component. However, the reduction in the area of the external concrete decreases its fire resistance, leading to a higher maximum fire temperature experienced by the core concrete and an increased degree of deterioration in the concrete material properties. As a result, the improvement in the ductility coefficient of the component is slightly attenuated.

#### 4.1.5. Influence of External Concrete Strength on Ductility

When the external concrete strengths are 30 MPa, 40 MPa, 50 MPa, and 60 MPa, the ductility coefficients of the components are 1.67, 1.68, 1.70, and 1.63, respectively. The variation range of the ductility coefficient is only 4.12% as the external concrete strength changes, indicating that the external concrete strength has little effect on the ductility coefficient of the member. When the strength of the external concrete reaches 60 MPa, the ductility coefficient decreases. This decrease occurs because at this point, the stiffness of the external concrete increases with the increase in strength. Under the action of horizontal load, the deformation ability of the component when it yields weakens, and thus the ductility decreases.

#### 4.1.6. Influence of Reinforcement Ratio on Ductility

When the reinforcement ratios are 0.0079, 0.0125, 0.0195, and 0.0304, the ductility coefficients of the components are 1.62, 1.84, 1.68, and 1.63, respectively. As the reinforcement ratio increases, the ductility coefficient of the ST-RC columns exhibits an initial increase followed by a decrease. When the reinforcement ratio increases from 0.0079 to 0.0125, the ductility coefficient of the member rises. This is because the reinforcement embedded in the external concrete enhances the fire resistance of the external concrete as the reinforcement ratio increases. Consequently, the deterioration of the core concrete material is reduced, leading to an improvement in the ductility of the member. However, when the reinforcement ratio increases from 0.0125 to 0.0304, the ductility coefficient of the member decreases. At this stage, excessive reinforcement results in limited deformation capacity at yield, and the reinforcement fails to yield when the external concrete crushes, thereby reducing the ductility of the member.

#### 4.1.7. Influence of Load Ratio on Ductility

When the load ratios are 0.2, 0.25, 0.3, and 0.35, the ductility coefficients of the components are 1.67, 1.68, 1.69, and 1.68, respectively. As the load ratio rises, the maximum variation in the ductility coefficient of the member is only 2.26%. Within the load ratio range of 0.2–0.35, its influence on the ductility coefficient of the member is minimal. This is because, although the load acting on the member increases with the load ratio, the increase is small and does not cause significant damage to the member, resulting in only minor changes in the ductility coefficient.

Based on the above analysis of the ductility coefficient of ST-RC columns after fire on one side, five key parameters, including heating time, slenderness ratio, section size, core area ratio, and reinforcement ratio, are selected as fundamental variables. Considering the correlations among these influencing factors and selecting a functional form with a high degree of fit, each parameter and the ductility coefficient are established as a matrix, and mathematical regression is conducted using 1stopt. The following formula for the ductility coefficient of ST-RC columns after fire on one side under cyclic loading is shown as Equation (3):

(3) $$\mu =\frac{(740.5199\rho_{b}^{2}-709.564\rho_{b}-30.9555)\times \lambda^{12.8468}\times B^{6.116}}{e^{7.4607T}\times \alpha_{sc}^{18.1534}}+1.7132$$

**Figure 17:**
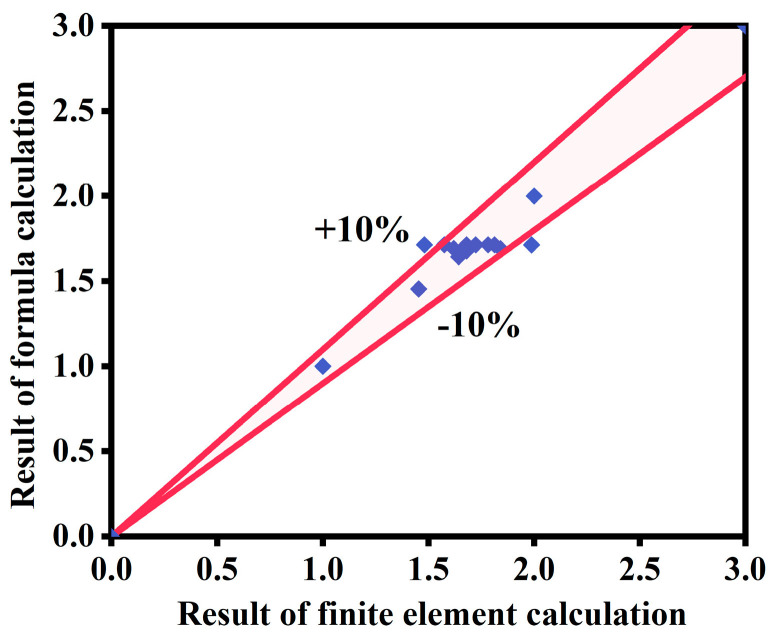
Comparison of simplified formulae and finite element calculation results for ductility coefficient.

This formula is assumed to be a numerical calculation and has no dimensional units when calculated. The values of each parameter are determined according to common units. The units for heating time, section size, and external concrete strength are minutes (min), millimeters (mm), and megapascals (MPa), respectively. Figure 17 presents a comparison between the calculated ductility coefficients of ST-RC columns under cyclic loading after fire on one side and the values predicted by the proposed formula. The comparison results demonstrate that the overall error between the two remains within 10%, confirming that the formula is highly consistent with the finite element calculations. This validates the formula’s ability to accurately determine the ductility coefficient of this type of component under specific conditions.

### 4.2. Energy Dissipation Capacity

#### 4.2.1. Influence of Heating Time

**Figure 18:**
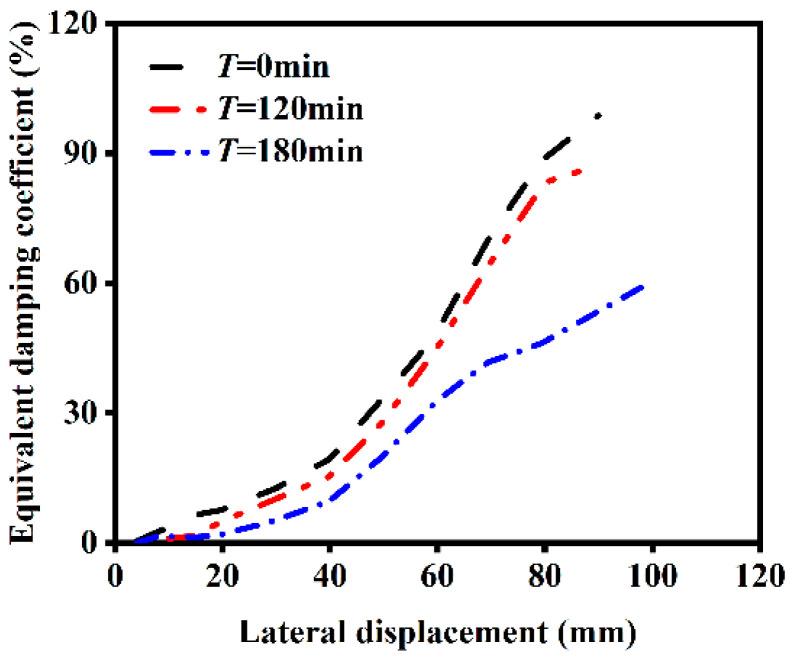
Effect of heating time on energy dissipation.

The influence of different heating times on the ST-RC columns after fire on one side is shown in Figure 18. As shown in the figure, as the heating time increases, the energy dissipation capacity of the component decreases after fire exposure on one side. This occurs because fire exposure weakens the material properties of the component, leading to degradation in strength and stiffness, and a reduction in energy dissipation capacity. Consequently, longer heating durations result in smaller equivalent viscous damping coefficients for the component. However, as horizontal cyclic loading progresses, the damage to the column becomes more severe, causing it to enter the elastoplastic stage earlier. This enhances the energy dissipation capacity of the component with continued loading.

#### 4.2.2. Influence of Slenderness Ratio

**Figure 19:**
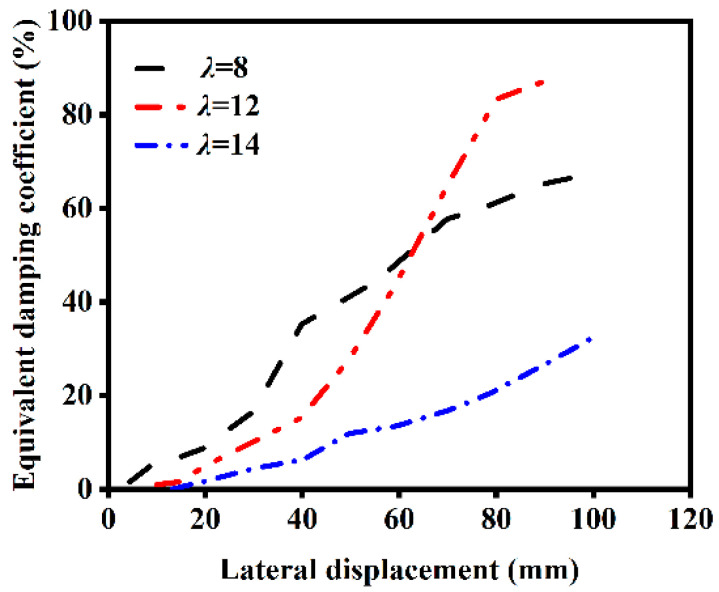
Effect of slenderness ratio on energy dissipation.

The influence of different slenderness ratios on the ST-RC columns after fire on one side is shown in Figure 19. As the slenderness ratio increases, the energy dissipation capacity of the component decreases after fire on one side. This occurs because at smaller slenderness ratios, the initial stiffness of the component is higher, leading to faster damage accumulation under horizontal cyclic loading. However, due to the protective effect of the core concrete, components with larger slenderness ratios do not experience destabilization. Instead, the equivalent viscous damping coefficient increases as damage progresses.

#### 4.2.3. Influence of Section Size

**Figure 20:**
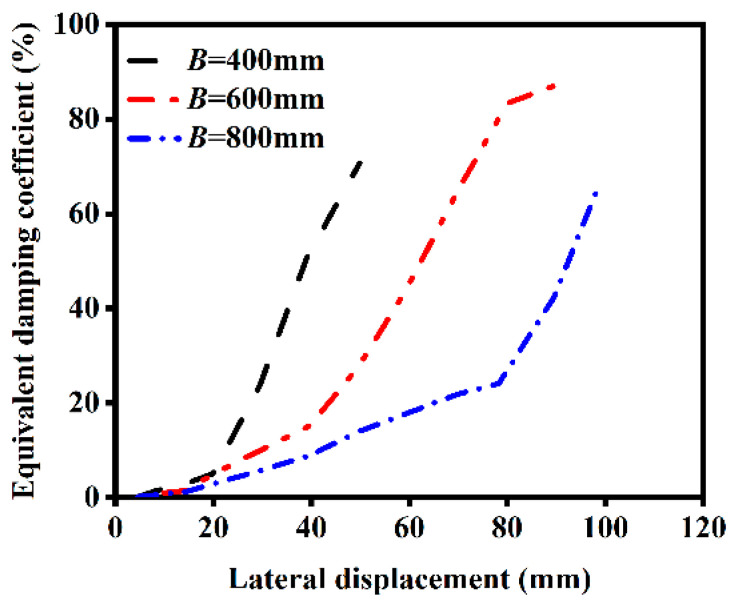
Effect of section size on energy dissipation.

The influence of different section sizes on the ST-RC columns after fire on one side is shown in Figure 20. As the loading displacement increases, larger section sizes result in a weaker ability to withstand earthquakes in the component. With the increase in section size, the deformation capacity of the component at yield is reduced, the stiffness becomes higher, and the rate of damage accumulation accelerates under horizontal cyclic loading. Consequently, the energy dissipation capacity is diminished.

#### 4.2.4. Influence of Core Area Ratio

**Figure 21:**
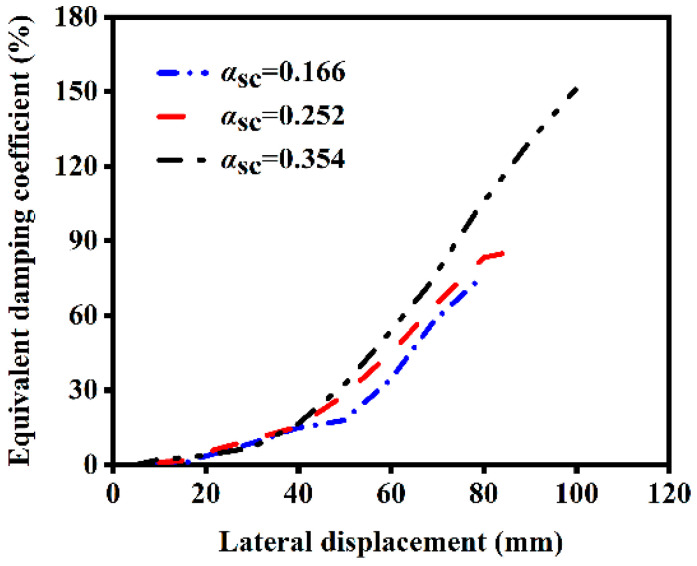
Effect of core area ratio on energy dissipation.

The influence of different core area ratios on the ST-RC columns after a single-side fire is shown in Figure 21. As the core area ratio increases, the energy dissipation capacity of the component gradually weakens after a single-side fire. This occurs because an increase in the core area ratio results in a reduction of the external concrete area. During single-side fire exposure, the core concrete experiences significant damage, leading to a weakening of the component’s energy dissipation capacity.

#### 4.2.5. Influence of External Concrete Strength

**Figure 22:**
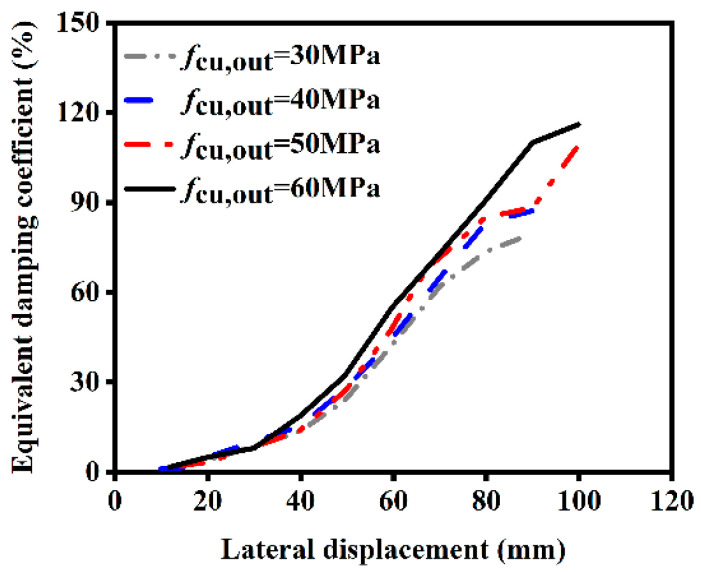
Effect of external concrete strength on energy dissipation.

The influence of different external concrete strengths on the ST-RC columns after fire on one side is shown in Figure 22. As shown in the figure, an increase in the strength of the external concrete leads to an improvement in the energy dissipation capacity of the member after fire on one side. This occurs because the external concrete possesses fire resistance capabilities; the higher its strength, the greater the fire resistance of the component, resulting in less damage to the core concrete. Consequently, the energy dissipation capacity of the component is slightly enhanced.

#### 4.2.6. Influence of Reinforcement Ratio

**Figure 23:**
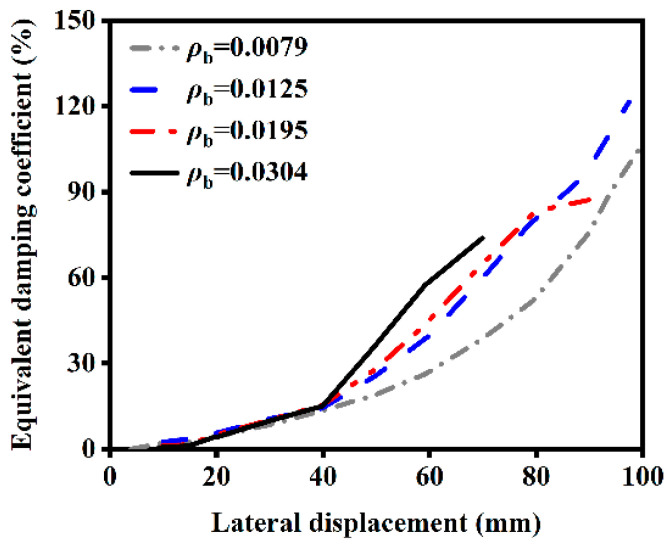
Effect of reinforcement ratio on energy dissipation.

The influence of different reinforcement ratios on the ST-RC columns after fire on one side is shown in Figure 23. As the reinforcement ratio increases, the energy dissipation capacity of the member after fire on one side gradually improves. This is because the reinforcement, embedded within the external concrete, enhances the bond between the concrete and rebar, thereby improving the fire resistance of the external concrete and reducing damage to the core concrete. Therefore, the energy dissipation capacity of the component is enhanced.

#### 4.2.7. Influence of Load Ratio

**Figure 24:**
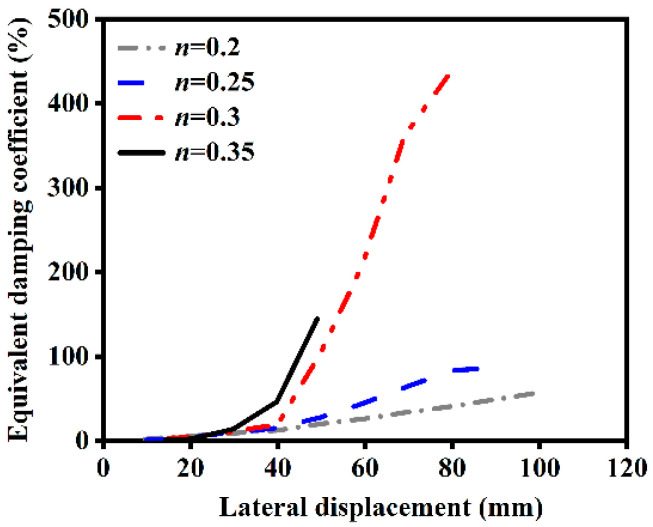
Effect of load ratio on energy dissipation.

The influence of different load ratios on the ST-RC columns after fire on one side is shown in Figure 24. As the load ratio increases, the energy dissipation capacity of the member after fire on one side gradually improves. This is because a higher load ratio results in a greater axial load, causing the member to enter the plastic deformation stage earlier under cyclic loading. Consequently, more energy is dissipated, leading to enhanced energy dissipation capacity.

Based on the analysis of the viscous damping coefficient of the ST-RC column after fire on one side, eight key parameters, namely, loading displacement, heating time, slenderness ratio, section size, core area ratio, external concrete strength, reinforcement ratio, and load ratio, are selected as the basic variables. Considering the interrelation of these factors, each parameter and the viscous damping coefficient are established as a matrix, and mathematical regression is conducted using 1stop. The coefficient formula for the viscous damping coefficient of the ST-RC column after fire on one side under reciprocating load is presented in Equation (4) as follows:

(4) $$h_{e}=0.0008\frac{f_{cu,out}^{0.4476}\times B^{0.0094}\times (0.0072\rho_{b}^{2}-0.7408\rho_{b}+281.9207)\times (-0.0201\lambda +0.0372)}{e^{8.2458\Delta }\times \alpha_{sc}^{-1.2962}\times T^{0.0555}\times {\left(0.2386n^{2}-4.8124n+26.8529\right)}^{-1}}-0.6464$$

**Figure 25:**
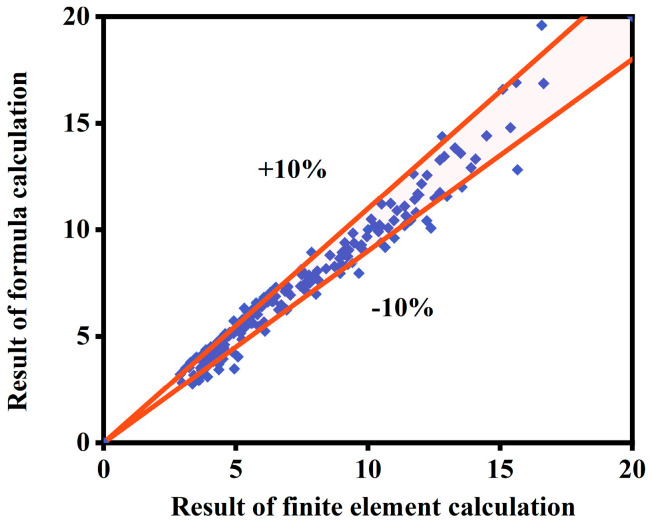
Comparison of simplified formulae and finite element calculation results for viscous damping coefficient.

This formula is assumed to be a numerical calculation and yields a dimensionless quantity when calculated. The values of each parameter are determined according to common units. The units for loading displacement, heating time, section size, and external concrete strength are millimeters (mm), minutes (min), millimeters (mm), and megapascals (MPa), respectively. Figure 25 presents a comparison between the calculated viscous damping coefficients and the values predicted by the formula for ST-RC columns after fire on one side under cyclic loading. The comparison results demonstrate that the overall error between the two remains within 10%, indicating strong agreement between the formula calculations and the finite element analysis results. This confirms that the formula can accurately determine the viscous damping coefficient of this type of member under the specified conditions.

## 5. Conclusions

In this paper, the seismic performance of ST-RC columns after fire on one side is analyzed in depth, and the following conclusions are drawn:

1. The ductility of ST-RC columns exposed to fire on one side will be weakened, and the ductility coefficient will be decreased by 5.62% compared with that of non-fire members, while the ductility coefficient of ST-RC columns exposed to fire on all sides will be decreased by 11.24% compared with that of non-fire members. It can be seen that the ductility of single-sided fire-exposed components is not significant, while the ductility of four-sided fire-exposed components is more significant, about twice that of single-sided fire-exposed components.
2. The initial stiffness of members exposed to fire on one side decreased by 24.69% compared with that of non-fire members, while the stiffness of members exposed to fire on all sides decreased by 69.37% compared with that of non-fire members. It can be seen that the greater the fire exposure, the smaller the initial stiffness, because the elastic modulus of the steel cannot be restored to its initial state after the fire.
3. The energy dissipation capacity of the component will decay after unilateral fire, and it will continue to increase during the the loading process. When the component enters the plastic deformation stage, the energy dissipation capacity will suddenly increase.
4. The heating time, slenderness ratio, section size, core area ratio, and reinforcement ratio are the main factors that affect the ductility of ST-RC columns after fire on one side. The heating time, slenderness ratio, section size, core area ratio, external concrete strength, reinforcement ratio, and load ratio are the main factors that affect the energy dissipation capacity of ST-RC columns after fire on one side.

## Figures and Tables

**Table 1:** Specimen basic parameters.

Scheme	Length (mm)	Diameter or Side Length (mm)	Pipe Outer Diameter and Wall Thickness	Reference
CCS1	1100	300	168 × 5.76	Qian [5]
CCS2	1100	300	168 × 5.76	Qian [5]
CF1	1500	133	133 × 4.7	Lin [24]
CF3	1500	133	133 × 4.7	Lin [24]
CF5-2	1500	133	133 × 4.7	Lin [24]
CF6	1500	133	133 × 4.7	Lin [24]

**Table 2:** Basic specimen parameters.

*B* × *D* × *t* × *L* (mm)	*e′*	*ρ_b_*	*n*	*f*_y_ (MPa)	*α*_sc_	*f*_cu,out_	*f*_cu,in_
600 × 360 × 10 × 7200	0	0.01946	0.3	390	0.252	C40	C60

**Table 3:** Characteristic parameters of the skeleton curve for each parameter.

Specimens	Ductility Coefficient	Specimens	Ductility Coefficient
*T* = 0 min	1.78	*λ* = 8	2.0
*T* = 120 min	1.68	*λ* = 14	1.45
*T* = 180 min	1.58	*α*_sc_ = 0.095	1.64
*B* = 400 mm	1.48	*α*_sc_ = 0.166	1.68
*B* = 1000 mm	1.72	*α*_sc_ = 0.354	1.81
*f*_cu,out_ = 30 MPa	1.66	*ρ*_b_ = 0.0079	1.62
*f*_cu,out_ = 50 MPa	1.7	*ρ*_b_ = 0.0125	1.84
*f*_cu,out_ = 60 MPa	1.63	*ρ*_b_ = 0.0304	1.63
*n* = 0.2	1.67		
*n* = 0.3	1.69		
*n* = 0.35	1.68		

## Data Availability

The original contributions presented in this study are included in the article. Further inquiries can be directed to the corresponding author.
